# Ugandan providers’ views on the acceptability of contraceptive self-injection for adolescents: a qualitative study

**DOI:** 10.1186/s12978-018-0611-7

**Published:** 2018-10-03

**Authors:** Jane Cover, Allen Namagembe, Justine Tumusiime, Jeanette Lim, Carie Muntifering Cox

**Affiliations:** 1 PATH, PO Box 900922, Seattle, WA 98109 USA; 2 PATH, PO Box 7404, Kampala, Uganda; 3 St Catherine University, St. Paul Campus, 2004 Randolph Ave, St. Paul, MN 55105 USA

**Keywords:** Self-injection, Injectable contraception, DMPA-SC, Depot medroxyprogesterone acetate, Adolescents, Family planning

## Abstract

**Background:**

Self-injection of subcutaneous depot medroxyprogesterone acetate may offer greater discretion and increase access to injectable contraception, particularly for those who face challenges accessing clinic services. In particular, unmarried adolescents often encounter stigma when seeking services, and may also lack the financial means to travel to clinics on the quarterly basis that injectable contraception requires. Whether self-injection is offered to women on a wide scale basis, and to adolescents specifically, will depend in part upon the willingness of providers to train clients of diverse ages and educational backgrounds. This study explores the views of providers with regard to self-injection as an option for women and adolescents in Uganda.

**Methods:**

In-depth qualitative interviews were conducted with family planning providers in Gulu district, to understand their views on injectable self-injection for women, with a specific focus on unmarried adolescents ages 15 to 19 years. The in-depth interviews, which lasted up to 60 min were audio-recorded, translated and transcribed simultaneously, and analyzed using Atlas.ti software to identify key themes and common perspectives.

**Results:**

A total of 40 health care providers were interviewed with equal numbers of each type (public, NGO, and private clinics, pharmacies, and community-based health workers). While most providers were receptive to self-injection for adult women, fewer than half were supportive of adolescent self-injection. Their reservations focused on age, marital status and parity concerns around adolescent use of the injectable more broadly, and concerns about the ability of adolescents to self-inject safely.

**Conclusions:**

Self-injection presents an opportunity to reduce the enormous burden on the public sector health system in Uganda, which is particularly compounded by the heavy reliance on injectable contraception requiring quarterly clinic visits. The results of this study reveal a level of cautious support for self-injection among providers when it comes to self-injection by adult women. With respect to adolescent clients, family planning policymakers and program implementers should design, implement, and evaluate self-injection interventions with the needs of adolescent clients uppermost in mind, recognizing that extra attention will likely be needed to reduce provider-imposed restrictions on adolescent access to this injectable delivery modality.

## Plain English summary

Self-administration of contraception may offer greater privacy and increase access to injectable contraception, particularly for those who face challenges accessing clinic services. In particular, unmarried adolescents often encounter negative stigma when seeking services, and may also lack the financial means to travel to clinics for regular reinjection. Whether self-injection is offered to adolescent women will depend on the willingness of providers to train clients of diverse ages and backgrounds. This study explores the views of providers with regard to self-injection as an option for women and adolescents in Uganda.

In-depth qualitative interviews were conducted with family planning providers in Gulu district, to understand their views on injectable self-injection for women, with a specific focus on unmarried adolescents.

A total of 40 health care providers were interviewed from public, NGO, and private clinics, pharmacies, and community-based settings. While most providers were receptive to self-injection for adult women, fewer than half were supportive of adolescent self-injection. Their reservations focused on whether young women, women who are unmarried, and women without children should use the injectable, in addition to concerns about the ability of adolescents to self-inject safely.

Self-injection presents an opportunity to reduce the enormous burden on the public health system imposed by quarterly reinjection clinic visits. These results reveal cautious support for self-injection among providers when it comes to self-injection by adult women. With respect to adolescent clients, extra attention will likely be needed to reduce provider-imposed restrictions on adolescent access to this mode of delivery for injectable contraception.

## Background

The introduction of subcutaneous depot medroxyprogesterone acetate (DMPA-SC) in a number of African countries in 2014 has opened the door to the possibility of self-injection of injectable contraception [1]. Self-injection may offer greater discretion and increase access to the injectable, particularly for those who face challenges accessing clinic services. Many women could benefit in terms of reduced travel and opportunity costs if they could manage their injectable use independently of the health system. In particular, unmarried adolescents often encounter stigma when seeking contraceptives at public sector clinics, and may lack the financial and physical independence to travel to clinics on the periodic basis that injectable contraception requires. Whether self-injection is offered to women on a wide scale basis, and to adolescents specifically, will depend on the willingness of providers as gatekeepers to DMPA-SC. This qualitative study explores the views of providers – public, NGO, private and community-based – with regard to self-injection as an option for women and adolescents in Uganda.

Like the intramuscular version, DMPA-SC is a three-month, progestin-only product that is stable at room temperature. The subcutaneous version now available in a number of African countries has a lower dose of DMPA (104 mg. vs. 150 mg.), but with comparable safety and efficacy [2]. DMPA-SC is packaged in the Uniject™ injection system – a small, prefilled, auto-disable device designed for easy administration after minimal training. The easy-to-use injection system provides opportunities for women to administer themselves through self-injection. DMPA-SC (brand name Sayana Press) was registered for administration by providers by the Ugandan National Drug Authority (NDA) in 2014.

While the total fertility rate in Uganda is declining – from 6.2 children per woman in 2011 to 5.4 in 2016 – it remains among the highest in the world [3, 4]. Unmet need for contraception is also high, at 28% among currently married women and 32% among sexually active unmarried women, suggesting that a substantial share of women are not using a method though they would like to wait an additional 2 years before their next birth or to limit childbearing altogether [5]. Adolescents aged 15–19 years represent about 11% of the population in Uganda, and one quarter of them have already begun childbearing [5]. The modern contraceptive prevalence rate is 36% among married women ages 15 to 49 years, and the injectable is the most popular method (for married and unmarried women alike), representing nearly half (46%) of the modern method mix [6].Studies of the appeal of DMPA-SC in Uganda found that more than four out of five women preferred it to the intramuscular version (DMPA-IM) [7]. The the country is now undergoing rapid scale up to offer DMPA-SC nationwide, and as of 2018, DMPA-SC represents 11% of the contraceptive method mix among married women [6].

The popularity of injectable contraception, and the growing appeal of DMPA-SC in Uganda creates potentially fertile ground for self-administration. Self-injection of DMPA-SC could overcome barriers for many women, and particularly for adolescents. While women may initially need training from a clinic- or community-based health worker, subsequent self-injection at home will eliminate the need to see a provider every 3 months. Self-injection reduces the financial burden and opportunity costs associated with travelling to the clinic and waiting for services. The ability to store the product at home will make women less vulnerable to stock outs. For women wishing to use the injectable discreetly, self-injection may enhance confidentiality. In short, self-injection may improve injectable continuation by reducing clinic access challenges while simultaneously enhancing women’s autonomy and control over contraceptive use [8–11].

Self-injection may offer solutions to the particular access challenges often faced by unmarried adolescents, including concerns about privacy and confidentiality stemming from the stigma of contraceptive use and premarital sex; the cost of travel and distance to health facilities for adolescents who lack financial autonomy and face challenges travelling independently; and inconvenient clinic hours, which can be particularly difficult for school-attending adolescents [12, 13]. Despite efforts to improve services for adolescents, there continues to be a pronounced lack of youth-friendly services, of which non-judgmental, supportive front line health care providers are the critical component [14, 15].

Numerous studies have found that health care workers sometimes refuse to provide contraception to unmarried adolescents because of deeply held negative opinions about premarital sex, or limit their contraceptive options due to misperceptions about who can use certain methods safely and effectively [13]. Misplaced fears that some forms of contraception impair fertility lead to parity and/or age restrictions [16, 17]. Recent research from Nigeria found that the most common restriction imposed on contraceptive provision was an age requirement [18]. In Tanzania, more than one in three providers impose age restrictions on injectable use, more than one in four impose parity restrictions and one in five impose marriage restrictions [19]. More specific to Uganda, a simulated client survey found that two thirds of providers chose a method on behalf of their clients. The authors observed that ‘younger clients seemed to be treated differently than older clients’, with more discussion focused on method side effects when the simulated client was younger and unmarried [20]. Another study from Uganda found that one in five providers indicated they would not offer injectable contraception to adolescents, with the authors concluding that most providers had misunderstandings about contraceptives, negative attitudes toward providing contraceptives to young women, and imposed age restrictions and consent requirements on adolescents [21]. While self-injection does not eliminate the challenge of provider bias, it improves the autonomy of clients and may enable unmarried adolescents to minimize the stigma they experience when seeking contraceptives in clinic settings.

Research suggests that self-injection is a feasible mode of administration, including for women in low resource settings as well as for adolescent women. Among adult research participants in Uganda, 88% self-injected proficiently three-months after being trained by a nurse [22]. Studies from high resource settings have similarly shown that self-injection of DMPA-SC is feasible [23–25]. One study in the United States specifically assessed adolescent competency and acceptability in self-administering DMPA-SC, finding moderate proficiency (63%) after a single training session. Though the study was small, the authors concluded that self-injection was feasible for adolescents with training and support [26]. A qualitative study of Ugandan adolescent interest in self-injection found that approximately half were personally interested in taking up self-injection if that option were available to them [27].

With respect to self-injection in Uganda, the NDA granted approval for self-injection in 2017, and subsequently that year, a pilot self-injection program was launched in four districts – the first offer of self-injection in sub-Saharan Africa outside of a research setting. However, successful implementation and scale up of self-injection as a delivery strategy in Uganda will require buy-in from family planning providers who are the gatekeepers to self-injection training. To the extent that providers doubt women’s ability to self-inject safely and effectively, or feel that self-injection will encourage promiscuity among unmarried adolescents, they will limit the availability of training, or provide training only to particular types of women, such as more educated or married adult women.

This qualitative study is designed to better understand Ugandan providers' willingness to endorse and train women, including adolescents, for self-injection. To that end, we first explore providers’ views of appropriate contraceptive methods for adolescents, including injectable contraceptive use, as a necessary precondition to self-injection. The study then examines their views regarding self-injection for all women of reproductive age. Lastly it solicits their opinions regarding key program characteristics—such as the training approach and appropriate follow-up—that might facilitate self-injection, and how the program design requirements may be varied to better serve adolescent clients.

## Methods

### Study sites and participants

The study was conducted between October – December 2015 in the district of Gulu in Northern Uganda, in collaboration with the Ugandan Ministry of Health. Facilities and establishments from which participants were drawn were identified from a full list of potential sites in the district, and those selected were based on the willingness of facility administrators to have their staff participate, and with consideration for the accessibility of the site. Health care personnel were recruited purposively from private clinics, NGO clinics, public sector clinics, public sector village health teams (VHTs), and pharmacies. Clinic-based family planning providers and community health workers were eligible if they were providing family planning counseling and services as part of their main responsibilities, while pharmacy staff were eligible if their pharmacy was selling  injectable contraceptives. Participants were recruited through face to face interaction at facilities and interviews conducted either at the facility or at a location convenient to the provider. Participants were required to speak English or Acholi—the major local language, to provide voluntary informed consent and to agree to being audio-recorded.

### Study design and procedures

We conducted qualitative in-depth interviews with family planning providers to understand their views on injectable self-injection for women, with a specific focus on unmarried adolescents ages 15 to 19 years. Participants included a mix of individuals who had previously been trained to administer DMPA- SC, and others who were unfamiliar with DMPA-SC. Health workers who had not been trained to administer DMPA-SC were given basic information, received a demonstration during the interview, and had an opportunity to administer DMPA-SC on a prosthetic. No injections or self-injections were performed during this study.

Semi-structured interview guides were developed to facilitate the interviews. Different interview guides were prepared for family planning providers who had previously been trained to administer DMPA-SC, family planning providers not familiar with DMPA-SC, and with pharmacy staff.

The data collection team was comprised of both male (2) and female (2) research assistants from Gulu district. They participated in a 5-day training covering recruiting and screening procedures, research ethics and administering informed consent, qualitative interviewing techniques and conducting in-depth interviews, translating and transcribing audio recordings, and data quality assurance.

The in-depth interviews lasted up to 60 min and were conducted in English or Acholi, as preferred by the participant. Interviews were audio-recorded, and translated and transcribed simultaneously.

Interviews were designed to move from the general to specific, beginning with views on contraceptive use and recommended methods for women in general, and for adolescents specifically, and progressing to views about injectable contraception and self-injection as a delivery modality for adult as well as adolescent women. Interviewers also solicited ideas for program design to facilitate self-injection—such as training, supervision, and reminders – with an eye toward what additional support, if any, might be necessary for adolescent populations.

### Data analysis

Data analysis was conducted using the qualitative software program Atlas.ti. The coding scheme was developed and transcripts were coded through an iterative process by two coders, with any discrepancies reviewed and resolved. Coded text was reviewed for each main code and, subsequently, memos were developed to summarize key patterns and themes. Where appropriate, findings were compared by the type of provider (private clinic/public clinic/commercial provider/community based).

### Ethical conduct of the study

All research study team members involved in data collection, management, or analysis were trained on research ethics, including confidentiality. This study was approved by the Mulago Hospital institutional review board, the Uganda National Council for Science and Technology, and the PATH Research Ethics Committee.

## Results

### Participant characteristics

To capture the views from various types of providers, we interviewed 40 providers in total, 29 of whom were women. The median number of years worked as a health care provider was 6, with a range of one to 30 years. The median age was 32, with a range of 24 to 75 years. To maximize the diversity of experience, providers were drawn equally from public sector health centers, NGO facilities, for profit clinics, pharmacies and the public sector community-based distribution program. Participant characteristics are shown in Table 1.

**Table 1:** Participant characteristics

	*N* (Total = 40)	%
Median age (and range)	32 (24–75)	
Gender		
Male	11	27.5
Female	29	72.5
Religion		
Catholic	22	55.0
Protestant	12	30.0
Pentacostal	4	10.0
Seventh Day Adventist	2	5.0
Facility		
Private clinic	9	22.5
Pharmacy	8	20.0
Public Community Health Worker	8	20.0
Public clinic	8	20.0
NGO clinic	7	17.5
Title/Background		
Clinical officer	5	12.5
Midwife	7	17.5
Nurse/Nursing officer	14	35.0
Nursing assistant	4	10.0
VHT (Community Health Worker)	8	20.0
Pharmacist	1	2.5
Business manager	1	2.5
Median number of years as health worker (and range)	6 (1–30)	

Below we present summaries of themes that emerged from interviews, along with illustrative comments from these 40 health care providers.

### Informed choice for adult women, less so for adolescents

When asked what family planning methods they recommend to a client, about three fourths of providers (*n* = 28) stated that they do not recommend specific methods, but rather, counsel the woman about available family planning methods and allow her to choose.

*“I do not decide for the mothers any methods. But when they come, I counsel them and then they choose for themselves.” – VHT provider*

The remaining providers who stated that they recommend particular methods were evenly split between those who recommend injectables and those who promote long acting reversible methods and sterilization (intrauterine devices or IUDs, implants and tubal ligation). No one offered that they recommend condoms or oral contraceptives.

With regard to unmarried adolescents under age 20 however, far fewer providers (*n* = 14) expressed that all (non-permanent) methods are potentially appropriate for adolescent women. The most common method actively promoted to adolescents was the condom (*n* = 18). Two thirds of providers recommending condoms (*n* = 12) were concerned about exposure to sexually transmitted infections (STIs), and the remaining one third (*n* = 6) expressed reservations about premarital adolescent sexual activity, recommending the ‘ABCs’ – Abstain, Be faithful but if not, use Condoms.

*“The method that I feel is appropriate for an adolescent according to me is only condom. Because like for the female adolescent there are side effects of these contraceptives and it can become problematic especially to those who are school going. So condom is okay since it doesn’t only prevent them from pregnancy but also from other diseases like the STDs (sexually transmitted diseases), for instance HIV/AIDs (Human immunodeficiency Virus/Acquired Immune Deficiency Syndrome).”* - Pharmacy provider

*“But you know when we talking about adolescents, we are not only looking at preventing pregnancy in them but also other STDs and STIs. Like adolescents who have not yet given birth in their life, as long as they know they are free from getting pregnant, they are very vulnerable and they would forget about HIVs. That would also be my fear that much as we are trying to prevent pregnancy in especially adolescents, we should always not forget to encourage them to use condom on top of preventing pregnancy. Remind them that HIV is still there, so they should protect themselves.”* - NGO provider

“*Being an adolescent is really not something easy because we always advocate for abstinence, you see that. And whoever cannot really do that should use condom.”* (Public provider)

Consistent with this tendency to prescribe specific methods to adolescent clients, a substantial share of providers (*n* = 15) volunteered specific methods they would advise adolescents NOT to use. In particular, they singled out the IUD (*n* = 4), hormonal methods generally (*n* = 2), injectable contraception (*n* = 3), cycle or moon beads (*n =* 2), long term methods of any type (*n =* 1), oral contraceptives (*n =* 1), implants (*n =* 1) and condoms (*n =* 1). Their rationales for restricting adolescent choice included concerns about low efficacy (condoms and cycle or moon beads), concerns about possible infertility or cervical cancer due to STI exposure during IUD use, and more commonly, concerns about the impact of hormonal contraception on fertility.

### Providers divided on injectable use by adolescents

When asked specifically about injectable contraception, just over half of providers (*n =* 22) expressed support for adolescent use of this method, with many citing the importance of injectable contraception in preventing unwanted pregnancy and reducing the incidence of school drop due to pregnancy. Other stated benefits of the injectable included the short duration, manageable side effects, and accessibility of the method.

*“I would [offer injectable contraceptive to adolescents] because first of all I know it has no other future dangerous effect to the youth. Secondly it’s going to protect them from having unwanted pregnancy. And they will also continue with their studies at school. That is the reason I recommend the injectables to the youth. It’s very safe and it has no future effect.”- Public provider*

*“My own opinion is generally that it’s actually good for adolescents to use injectable contraception because the injectable is a short term method, and the side effects are easily manageable and it can easily be accessed and administered.”- NGO provider*

The remaining providers (*n* = 18) expressed reservations about adolescent use of the injectable. Their reservations were sometimes tied to marital status, rooted either in the perceived immorality of premarital sexual activity or in the risk of exposure to STIs through multiple sexual partners.

*“Assuming the adolescent is not yet twenty years, and is even not mature enough to marry, I think it would give them too much sexual feelings because she would -- after all if I inject this -- I don’t have any chance of pregnancy. So it would give them too much feelings to go for sex, yet they are still an inappropriate age to be having sexual intercourse.” - VHT provider*

*“I don’t feel comfortable at all [offering injectable contraceptives to adolescents]. Being a health worker and at the same time a mother, I would offer [injectables] because I will be thinking of the future of this girl as important. But again on the other side, I will be having feelings in my heart that am I not pushing this girl to make a mistake because she can now think I cannot conceive so I can do anything at any time.” – Public provider*

*“Adolescents who are married, they are already living as husband and wife; it (the injectable) will be good for them. But for those ones who are not married, the bad part of it is that they will now not fear HIV and hepatitis B. Because what I know about youth, they fear pregnancy. They just fear to get pregnant. Now if there are these methods of injectables which are there for adolescent, if they use it, they will forget that I should use protection during sex because I may acquire HIV or Hepatitis B. You see, that is the bad part of it.” – Pharmacy provider*

More common however, were reservations related to parity, rooted in the misconception that the injectable causes infertility, which has origins in the delayed return to fertility common among DMPA users.

*“The injection Depo is not recommended for the adolescent because it may interfere with their fertility since they have not produced in their life. So when it comes time for you to conceive, there may come a problem because the Depo takes a time long to leave the body system.” – Private provider*

*“The young ones, we always advise them to use oral contraceptives because inject-a-plan [brand name of the socially marketed injectable] is meant for once you have at least three or four children. It can stop you from getting your normal menstruation period.” – Private provider*

*“When they are married and the person has not yet delivered, it (the injectable) is not very advisable.” – Pharmacy provider*

Consistent with their reservations, more than one third of clinic-based providers felt that parental permission should be required (or is advisable) before offering family planning services to adolescents.

*“Adolescents who are not married, I think parental permission for adolescents to use family planning is appropriate. The parent should be aware because as those people who use family planning methods say, methods have side effects. So if it starts before the parents, when she has no knowledge, the parents could have fears about what is happening.” – VHT provider*

### Subcutaneous DMPA popular among providers

With respect to DMPA-SC specifically, more than three quarters of participants (*n* = 33) found the device easy to use, with the same number indicating they preferred it to intramuscular DMPA (DMPA-IM). Interviewees noted that the pre-packaged, all-in-one presentation offers advantages to providers, such as avoiding stock-outs of syringes, requiring less skill to administer, saving health workers’ time, and minimizing the risk of a needle stick.

*“It has its needle already attached. I don’t have to withdraw the Depo or the medicine. So everything about it is easier or much better or more convenient.”– NGO provider*

“*As I said, this issue of we don’t have the syringe is not there because everything is connected and you can administer it by yourself. It does not really require much skill in injecting. Actually there is nothing I don’t like about that.”– NGO provider*

Providers also identified benefits of DMPA-SC for women, including that the presentation readily lends itself to self-administration (*n* = 21), injections may be less painful due to the smaller needle (*n* = 6) and the perception that the lower dose DMPA-SC has fewer side effects (*n* = 4).

*“I saw the needle was really very tiny, you don’t experience much pain compared to the other injectable, and the (DMPA-IM) needle is a bit big.” – NGO provider*

*“I think the product is very good because it is self-administered by the clients and side effects are not really serious like for other methods.” – Public provider*

*“For me what I have liked about this [DMPA-SC], the first thing, it is private. You can use it privately. Secondly, the needle is very small for people who fear injections. Then you can do it even self-injection. Actually there is nothing, for me I feel there is nothing wrong with this.”- Public provider*

### Most providers receptive to self-injection, perceive advantages for women

To gauge receptivity to self-injection, providers were asked whether they consider self-injection to be a good option for Ugandan women. By a margin of just under two to one, providers viewed self-injection favorably (*n* = 25), citing the benefits of greater convenience and time savings (*n* = 11), reduction in transport costs (*n* = 9), and better adherence to the reinjection schedule (*n* = 5). Two providers noted that offering self-injection may reduce provider workload. Even providers less receptive to self-injection readily identified advantages for women.

*“Mostly I think the major part of the population in Uganda they are below poverty line so if someone can reduce the cost of transport, and get the injectable contraceptive which can be administered by themselves, it can really give them some [savings].” – Pharmacy provider*

*“In my location, I think it’s going to be a good idea to help out women because most of them, during the season for digging (farming) they go really far away; we’ve had incidences that they miss out on their shots just because they were not close to the clinic. So if they have this, if they have Sayana Press with them, I think they can be able to carry it with them and inject themselves.”- NGO provider*

*“Sometimes in the health centers you can go and you find that the health worker is not yet there, but if you are self-injecting yourself and you have got it enough with you at home, you will be using it without again need to look for health worker to help you inject you, all that.”- Pharmacy provider*

A number of providers (*n* = 11) spontaneously offered that self-injection is likely to enhance discretion for women who are hiding contraception use. When queried specifically about whether self-injection would be more discreet, nearly all the remaining providers (*n* = 27) thought self-injection would improve privacy.

*“I think all the women should use [self-injection] because there are some men who don’t advise their women to use family planning or to use any method. So Sayana Press can help them. You can even go to your private room and just inject yourself.”– Private provider*

*“Somebody who is concerned, maybe a neighbor, would say ‘I saw your wife at the facility, is she sick?’ You see? So it can bring problems.”- Private provider*

### Even receptive providers raise concerns

For a minority of providers (*n* = 12) however, their initial reaction to the concept of self-injection was more skeptical. These providers, as well as a number of providers generally receptive to self-injection, cited a number of concerns, including appropriate hygiene (*n* = 13), storage (*n* = 11), and disposal (*n* = 6) practices.

*“Normally those things have sterile procedures. If maybe the clients are not very clean the site may be infected.”– NGO provider*

*“Storage at home. I could be having the kitchen where it is hot throughout the day. I think we have a temperature where we should keep this Sayana Press. When I have only one house, it’s my bedroom, it’s my storage, it’s my kitchen. So it’s like we are exposing this Sayana Press at very high temperature which can damage the product.”– NGO provider*

*“What I see about this Sayana Press is convenient except the ways of disposing the waste. If it is not properly disposed, it can cause injuries at homes.”– Public provider*

In particular, some providers expressed a lack of confidence in women’s ability to administer the injection properly. This included opinions that women would forget the injection steps or not do them correctly (*n* = 11), forget their reinjection date (*n* = 4), or choose an inappropriate injection site (*n* = 5).

*“[Self-injection] is not so good because women are… they are not capable, intelligent enough to follow all the steps…It is better they go to the clinic or to the health workers to administer, not from home.” – Private provider*

*“These women if you put your mind on learning the steps in self-injecting and if the health provider did not write for you your next date of injection, you may forget since you may be too busy doing house work... But if you are to remember it alone, you will forget due to so much house work that women usually do at their homes.” – VHT provider*

*“And sometimes they may confuse the injection site. You see the drug has to be injected on the fatty tissue of the body, on the thigh and lower abdomen. So they can forget and end up injecting the wrong site and this may not be good for them.”– VHT provider*

Public sector clinic providers were disproportionately represented among the 12 individuals who were more skeptical about self-injection, with five of the eight public sector clinic providers expressing reservations. Conversely, public sector VHTs were disproportionally represented among those viewing self-injection favorably, with only one of eight VHTs expressing skepticism.

### Mixed feelings about adolescent self-injection

Regarding self-injection for adolescents, a slight majority of interviewees (*n* = 23) opined that self-injection was a good or acceptable option for adolescents—although 7 of these had previously stated that they did not approve of giving the injectable to adolescents. In particular, providers cited the specific benefit of enhanced privacy and ability to conceal contraceptive use (*n* = 21), which some felt, was particularly important for adolescents who are attending boarding school.

*“Because I think being in the health facility and also going to a family planning clinic, first of all [adolescents] fear being seen as I told you by the relatives, or the neighbors. But if they are now doing self-injection, they are more confident that nobody is seeing me.”– Public provider*

*“I think Sayana Press would be very good for the adolescents since they can even go with it to school and administer without anybody noticing. Because adolescents go to the boarding school far away from home and the drug will cover them even if they come back home for holidays.” – Public provider*

Sixteen interviewees felt that adolescents should not be permitted to do self-injection. Beyond concerns about injectable use by adolescents, the most common reason for opposition was a belief that adolescents do not have the maturity to do the injection on their own. Others worried that self-injection, like use of contraceptives more generally, would lead to sexual promiscuity, and a few were concerned that self-injection would open the door to illegal injectable drug use.

*“And for adolescents, they have to go to the health facility in order to get the injection because adolescents like taking things for granted. They may forget the procedure to follow when administering ...”– Private provider*

*“So someone like that (an adolescent), it is not appropriate for her because she might be given a method and she changes her mind to use another method. So the other method which she already had could expire unused. That is why I think that it (self-injection) is not very appropriate for adolescents.”– VHT provider*

*“The adolescents will misuse the drug. Misusing, I mean like I had live sex (without a condom) and maybe I can get pregnant, so I will also give the injection again.”– Pharmacy provider*

*“For the adolescents it would be beneficial but at the same time it would also cause a fear that once they get used to self-injection they may be introduced to some other drug like the drug use injection.”– Private provider*

Despite their general receptivity to self-injection (noted above), more than half of the VHTs (5 of 8) expressed reservations or concerns about self-injection by adolescents.

With regard to whether adolescents need parental permission to self-inject, providers were evenly divided with half indicating parental permission is needed and half indicating it is not. The rationales offered were similar to those for family planning use in general by adolescents.

*“I would think the adolescent should get permission from her parents so that in case of any risks in future the parents would be aware. Because if she starts doing it on her own, if she starts injection on her own, without informing her parents, if the side effects like bleeding starts, who will help her?” – Public provider*

*“Some of the parents they are very tough. Yes, because if you tell your parents I’m injecting myself and obviously the parents will know that you have somebody somewhere. And some of them may even stop paying your school fees when, if you are still at school.” – Pharmacy provider*

### Clinic providers best for training women

The vast majority of providers, regardless of the type of provider, identified clinic-based health workers as the most appropriate personnel to train women for self-injection, due to their medical expertise and contraceptive knowledge (*n* = 33).

*“Health workers are the most appropriate to train because there is a bit of medical knowledge involved in the injection and involved in knowing about the drug. Medical personnel may advise in an event that there’s anything like swelling or maybe infection at the site which has not been administered well. A medical personnel can also give and can handle that.” – Private provider*

*“Midwives who handle matters of family planning in the health center and in the area are appropriate and medical personnel with the knowledge on family planning.”– VHT provider*

That said, quite a few respondents recognized VHTs as capable of training women (*n* = 15), including nearly one third of those who first proposed clinic-based health workers (*n* = 10). VHTs are valued as trainers because of their close relationships with the community.

*“Like you heard me say, first, those people (VHTs) are prior trained and have knowledge on how to administer Sayana Press. Secondly, the VHT is a person closer and known to the community as a person from whom we get these specific things, from whom we go obtain this particular assistance.” – VHT provider*

While all eight pharmacists identified health workers as the most appropriate trainers, five indicated, when queried, that pharmacy staff could also train women for self-injection. One disagreed however, noting that pharmacists don’t have the time required to train women individually.

*“The health workers in the pharmacy should also be trained so that any clients going there, they should also give that knowledge to them... As you are selling the products to the customers, the health workers must know more about the product because you cannot give a product to a client and the client will ask you very many questions and you have nothing to say. The client will not take that unless you have knowledge on that, then you can give and defend with the answers.”– Pharmacy provider*

*“And the best people to train the women I would prefer those who work at the family planning section, maternity sites and antenatal care department because they are the ones who always interface with these women more often and they always carryout health education. Because like here at the pharmacy, no one can train these women since we provide a wide range of services and we do not have the time to talk to the clients for long.” - Pharmacy provider*

A few providers (*n* = 3) suggested that peer-to-peer training would be beneficial as it would provide role models to women.

*“I would recommend that there should be regular outreaches on Sayana Press and demonstration. And maybe select one participant to come and demonstrate by herself to see how they administer Sayana Press so that women get more knowledge and get acquainted with the method.”–NGO provider*

By about two to one, providers felt that adolescents might prefer or benefit from separate training—away from adults—for reasons such as discomfort and shyness. They opined that, if trained with older women they might not ask questions or receive adequate training. A few providers noted that HIV and STI education should also be a focus of training for adolescents (but did not mention these topics as critical for adult women).

*“The difference in the training needs maybe is there because there may be adolescents who are still very young like around 15 -16 years, and if you mix them with the older women, they may not ask any questions even if they have them, because of fear and not feeling free or comfortable.”– VHT provider*

*“[The training of adolescents should be] a little bit special because these people should be training them not only on family planning methods but also train adolescents on preventing themselves from being exposed to HIV/AIDs and other STIs. So this means your knowledge again will have to go further onto this adolescent. Those one who will be providing the services to the adolescent, they should equip them with more knowledge than these one who train mothers.”– Public provider*

### Post-training support and follow up important

About three quarters of providers (*n* = 28) mentioned that some form of proactive follow-up would be helpful to make sure clients remember the injection procedures and schedule. The most common form of follow up, identified by 19 interviewees, was home visits or community outreach to villages where women are self-injecting. Some specified that this form of follow-up would be most appropriate and feasible for the VHTs (*n* = 11). Others advocated that self-injecting clients be asked to return to the clinic periodically to demonstrate their mastery (*n* = 6). Some felt that phone calls to self-injecting clients would be appropriate support (*n* = 6)

*“I think it would be easy if we follow them through the VHTs that are nearer to them because if the VHT near them, this VHT can go to a mother just like he is visiting the home, and will follow up these mothers from home that is one thing. The VHT can also ask them how they are doing it and still remind them.”– Public provider*

*“Women, especially those ones in the villages, when you inject them or you tell them to always come back to you after a certain period, they do come. So according to me, I recommend that all providers should tell whoever gets Sayana Press from them to always remember to come back to him/ her after a certain period -- maybe after 6 months -- so that you can still assess the person to see whether she is doing it rightfully. In that way, you would also be doing refresher training.” – Pharmacy provider*

The remaining participants (*n* = 10) were confident that, once trained, women would not need additional supervision or follow-up to do self-injection. They advised that follow up should be client-initiated, such that women return to the facility or pharmacy in case of problems.

*“According to me, I feel if the women are cleared to start injecting from their homes, then they should tell them to always remember to come back to the health center in case they are forgetting the steps to follow in self-administering Sayana Press.”– VHT provider*

A number of providers recommended support in the form of a client instruction job aid and/or calendar be provided to remind women of the procedure and their next injection date (*n* = 8).

*“Yeah that one [client support] will be a little bit hard but I think they need to be encouraged to at least have a place where they can record the next date for injection. So they should be provided with either a calendar or a book where they can write their next injection date so that they cannot forget.”– Private provider*

Nine providers were of the opinion that follow-up was particularly fundamental for adolescents because they need more encouragement and supervision to continue with the method.

*“You know adolescent youth are not like adults. So for them they can change their mind anyhow so they need to be followed and talked with well. Advised.”– Private provider*

## Discussion

This study suggests that informed choice is an ideal not yet realized when it comes to contraceptive services for adolescent clients in Uganda. Our findings regarding what methods providers consider appropriate or inappropriate for adolescents are largely consistent with what Chandra-Mouli and colleagues refer to as a ‘condoms-only mindset’ [28]. Be they motivated by concerns about STIs, promiscuity, or lingering suspicions about the safety of hormonal methods, the attitudes of our participants suggest that providers may impose restrictions on method choice for young, unmarried and/or nulliparous adolescent clients. While providers may feel they have the adolescent’s best interests at heart in promoting condoms over other methods, adolescents may have older partners or engage in transactional sex, and may not be able to negotiate condom use in settings where gender norms promote inequality. Suspicion about the safety or appropriateness of high efficacy hormonal methods for adolescent women leaves them vulnerable to higher rates of contraceptive failure and method discontinuation associated with condoms and other short term methods [23, 29].

More specific to the main focus of this study, the parity and/or age restrictions on injectable use, which have no medical basis, present an obvious barrier to the offer of self-injection to this demographically-important population. Since fewer than half of providers see self-injection as a good option for adolescents, making self-injection available to adolescent clients will necessitate renewed efforts to overcome provider-imposed method restrictions and ease concerns about the ability of adolescents to self-inject safely. A number of promising interventions to reduce provider bias against adolescents have been proposed by the Beyond Bias Consortium, including: 1) clear directives and clarity from leadership regarding the importance of reproductive health services for adolescents; 2) provider education that involves communications training, values clarification, attitudinal conditioning or the pairing ‘reluctant’ providers with ‘champions’ in the provision of youth services, and using personalized anecdotes and exercises that encourage providers to see the world through an adolescent lens; and 3) a systems approach rather than one-off provider trainings to address the myriad conditions necessary for Youth-Friendly Services, such as expanded clinic hours, outreach to schools and communities, enhanced privacy, and reduced fees for adolescent clients [30].

With regard to self-injection for adult women, this study suggests that most providers view self-injection favorably, but with some reservations that will need to be addressed if the practice is to become widespread. In particular, provider concerns that women may be unable to self-inject competently should be addressed, with reference to the growing number of studies demonstrating its feasibility and acceptability, including for women in low resource settings and women with limited education [22, 31]. Identifying and featuring ‘self-injection champions’ is another strategy that may sway skeptical providers. Offering a client instruction guide and reinjection calendar may reassure health workers that women can self-inject independently. Concerns about safe storage and waste disposal may be assuaged by providing low cost impermeable containers to store the spent units until a convenient time to return the needles to a clinic, community health worker, or pharmacy.

With respect to other parameters for the design of a self-injection program, the vast majority of providers (of all types) identify clinic-based health workers as best placed to train women in self-injection. What providers may not recognize is that training women in self-injection can be time-consuming, particularly if training is conducted in a one-on-one fashion. The heavy workload of providers – particularly those in the public sector – may impinge on the availability of self-injection training. While less acceptable to providers in this study, permitting community-based health workers to train women may be a more realistic approach, and one that recent research suggests is feasible [8]. With regard to adolescents, a number of participants proposed that adolescents be trained separately from adult women, to reduce feelings of intimidation and discomfort. Making that proposal a reality likely entails offering self-injection training beyond the clinic, at outreach events or via programs that specifically target adolescent clients.

With respect to the private sector, though private clinic providers were not noticeably disinclined to endorse self-injection, it remains to be seen whether they will offer self-injection training, given their financial incentive to encourage repeat clientele in order to collect consultation fees. Training in commercial settings, such as pharmacies and drug shops, may be constrained by lack of a private setting for injections.

In terms of post-training support to self-injecting clients, most providers proposed proactive follow up to ensure that clients recall how and when to self-inject. This suggestion presents a number of challenges: First, many clients in Uganda are using methods discreetly, and much of the appeal of self-injection stems from the potential for enhanced confidentiality. In this setting, clients may not welcome a home visit or phone call from a family planning provider. Second, from a practical standpoint, the majority of women in Uganda do not have exclusive access to a cell phone, and providers are not resourced to provide home visits (or make calls to their clientele with phones). Tasking community health workers with follow up may be more practical and cost-effective, but will require strong coordination between clinic and field-based health workers. One option for client-initiated support currently being tested in Uganda is the offer of a toll-free hotline manned by trained self-injection counselors. If successful, this approach may satisfy the World Health Organization recommendation that self-injection be offered “in contexts where mechanisms to provide the woman with appropriate information and training exist, referral linkages to a healthcare provider are strong, and where monitoring and follow-up can be ensured” [32].

### Study limitations

As with all qualitative studies, our findings are not generalizable and may not apply to providers in other settings in Uganda and other countries. While attempts were made to solicit honest and forthcoming opinions, providers may have been subject to some degree of social desirability bias, offering opinions overly favorable to adolescent contraceptive use, injectable use, and/or self-injection.

## Conclusion

Self-injection presents an opportunity to reduce the burden on the health system presented by heavy reliance on injectable contraception requiring quarterly clinic visits. For adolescents, the potential discretion and user control inherent in the practice of self-injection align with well-established priorities for improving adolescent access to contraceptive services. While our results reveal a level of cautious support for self-injection among providers in Uganda, their reservations about offering the service to adolescents suggest more needs to be done if self-injection is to be made available to women without regard to age, parity or marital status. In addition to including self-injection in healthcare training curricula, service delivery guidelines will need to be revised and existing providers will require continuous professional development to bring them up to speed with this self-care intervention. With about a dozen countries in sub-Saharan Africa currently poised to introduce self-injection as a delivery option, this study offers insights on provider perspectives that ministries of health may wish to consider, particularly if their goals include improving access to family planning for adolescents. Moving forward, policymakers and program implementers should design, implement, and evaluate self-injection interventions with the needs of adolescent clients uppermost in mind, recognizing that extra effort may be required to shift provider perspectives and assuage their concerns.

## Abbreviations

AIDS: Acquired immune deficiency syndrome
DMPA: Depot medroxyprogesterone acetate
DMPA-IM: Intramuscular depot medroxyprogesterone acetate
DMPA-SC: Subcutaneous depot medroxyprogesterone acetate
HIV: Human immunodeficiency virus
IUD: Intrauterine device
NDA: National drug authority
NGO: Non-governmental Organization
STD: Sexually transmitted disease
STI: Sexually transmitted infection
VHT: Village health team
